# Conformational IgE Epitope Mapping of Der p 2 and the Evaluations of Two Candidate Hypoallergens for Immunotherapy

**DOI:** 10.1038/s41598-018-21792-1

**Published:** 2018-02-21

**Authors:** Kavita Reginald, Fook Tim Chew

**Affiliations:** 1 Research Centre for Biomedical Sciences, Sunway University, Bandar Sunway, 47500 Selangor, Malaysia; 2 Department of Biological Sciences, Sunway University, Bandar Sunway, 47500 Selangor, Malaysia; 3 Department of Biological Science, Allergy and Molecular Immunology Laboratory, National University of Singapore, Singapore, 117543 Singapore

## Abstract

Epitope mapping of Der p 2, a clinically important dust-mite allergen is the first step in designing immunotherapy hypoallergen vaccine candidates. Twenty-one single alanine mutants of Der p 2 were generated and their secondary structure was analysed using circular dichroism spectra. Only one mutant, K96A resulted in a misfolded protein. All mutants were tested for serum IgE reactivity using serum from dust mite allergic individuals by immuno dot-blots. Mutations to five residues, N10, E25, K77, K96 and E102 consistently showed reduced IgE reactions compared to wild-type Der p 2, and therefore these residues constitute the major IgE epitopes of Der p 2. Two mutants with consistent low IgE binding, K96A and E102A, were subsequently evaluated as hypoallergen candidates. IgG antibodies raised in mice against both mutants could inhibit human IgE-binding to WT Der p 2. Both mutants had intact T-cell epitopes as they were able to stimulate peripheral blood mononuclear cell proliferation similar to WT Der p 2. However, a switch in Th1:Th2 cytokine profile was not observed. In summary, we have identified the major conformational epitopes of Der p 2, and evaluated two Der p 2 hypoallergen vaccine candidates for immunotherapy.

## Introduction

About 30% of the world population suffer from allergic-related diseases and its prevalence is increasing annually^1^. House dust mites are the most important source of allergens in the indoor environment^2^, causing sensitizations in up to 90% allergic patients^3,4^. Currently 34 allergens originating from dust mites have been published (www.allergen.org). Of these, group 1 and 2 allergens, mainly from *Dermatophagoides spp*. are considered as major allergens, as they are recognized by more than 80% of the IgE from dust-mite sensitized individuals^4^.

An allergic response occurs when allergens bind and crosslink IgE antibodies on the surfaces of mast cells and basophils via the FcεRI receptor, causing these cells to get activated and release inflammatory mediators such as histamine, leukotriene and cytokines. Exposure to dust mite allergens can be managed by reducing physical exposure using exclusion methods such as dust-mite impermeable bedding covers and particulate air filters, or by managing symptoms using topical steroids or anti-histamines^1^. However, these measures are cumbersome and not long lasting.

The only curative treatment for allergies currently known is by allergen specific immunotherapy (SIT)^5^. Allergen SIT is based on repeated injections of increasing doses of allergen extracts and has been reported to have lasting effects, up to 3 years after discontinuation of treatment^6^. Conventionally, immunotherapy was performed using crude allergen extracts. This method risked allergic side effects, including anaphylaxis which could be fatal. To improve the safety of SIT, biologist are generating modified allergen proteins which have lesser IgE binding capacity, but retained T-cell proliferation^7^. Maintaining T-cell proliferation is important as studies on the mechanisms of immunotherapy have highlighted the role of allergen-specific CD4^+^ T-cells in tolerance mechanisms and control of allergic responses^8^.

In this study, we identify the conformational IgE epitopes of Der p 2 using site-directed mutations, and test these mutants for their reduction in IgE-binding capacity using serum from dust-mite allergic individuals. We then test two site directed mutants of Der p 2 for their potential as prophylactic hypoallergen vaccine in a murine model.

## Results

### Generation of single Der p 2 alanine mutants

**Figure 1:**
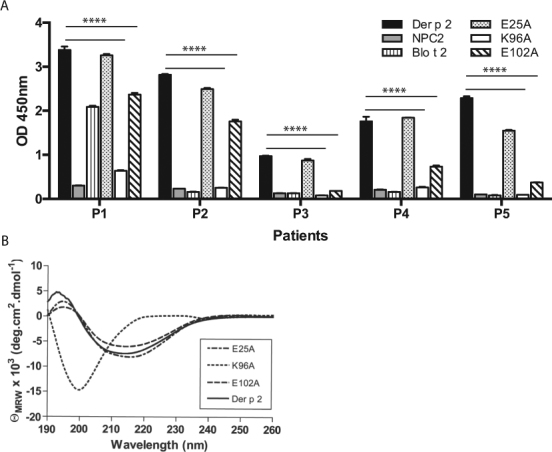
(**a**) Amount of IgE binding of 5 allergic individuals to Der p 2, hNPC2, Blo t 2 and selected site directed mutants of Der p 2. Mean and standard deviations of two independent assays performed in triplicates are presented. Differences between individual serum IgE binding to Der p 2 and mutants K96A and E102A were statistically significant (p < 0.0001). (**b**) Far UV circular dichroism spectra of wild type Der p 2, and mutants E25A, K96A, and E102A. Average spectra from 10 scans are shown.

In a pilot study, we evaluated the serum IgE of five Der p 2 allergic individuals to its structural homologue, human Niemann-Pick C2 protein (hNPC2), and found that hNPC2 did not show IgE binding (Fig. 1a). Therefore, we rationalized that the surface residues of Der p 2 that were different from hNPC2 most likely contained the IgE binding epitopes. Next, residues with similar side chain properties (Gln-Glu, Asp-Asn, Ser-Thr and Val-Thr), and those with very small side chains such as glycines and alanines were excluded as these residues were normally not involved in IgE interactions^9,10^. For the remaining surface residues, their side chain surface accessibility was calculated using the WHATIF algorithm^11^ based on the solved crystal structure of Der p 2^10^, as these residues were more likely to participate in protein-protein interactions^9^. Based on these considerations, ten residues with the highest side chain solvent accessibility and with different chemical properties from hNPC2 were selected for epitope mapping of Der p 2. We also included mutants of 11 residues which were previously reported to be involved in the IgE epitope of Der p 2^12–15^. Each residue was mutated to alanine and expressed as a recombinant protein (Table 1).

**Table 1:** Summary of IgE reactions of house dust mite allergic individuals to Der p 2 site directed mutants based on sensitization profiles.

No.	Mutants	Sensitization profile				Total
		Der p 2	Der p 2, Blo t 2	Der p 2, hNPC2	Der p 2, Blo t 2, hNPC2	
1	K6A^#,15^	*	*			2
2	**N10A**	*	*	*	*	**4**
3	H11A		*			1
4	K15A^a^	*	*	*		3
5	H22A	*	*			2
6	**E25A**	*	*	*	*	**4**
7	H30A^15,12^	*	*			2
8	K55A	*	*	*		3
9	L61A	*	*			2
10	E62A^15^	*	*			2
11	H74A^15^	*	*	*		3
12	**K77A**	*	*	*	*	**4**
13	Q85A		*			1
14	K89A	*	*			2
15	N93A^12^	*	*			2
16	**K96A** ^**12**^	*	*	*	*	**4**
17	I97A	*				1
18	K100A^14^	*	*	*		3
19	**E102A** ^**12**^	*	*	*	*	**4**
20	N114A^13,#^		*			1
21	R128A	*	*	*		3

Superscripts describes residues that has been previously described in other publications. The areas marked with asterisks (*) indicate that the IgE binding to individual mutants were < 75% compared to WT Der p 2 in at least 40% of the population. Mutants in bold had reduced IgE binding across all sensitization profiles. ^#^Residues which were similar in Der p 2 and hNPC2, but selected as they reported as IgE epitopes in previous studies.

All mutant recombinant proteins showed similar β-sheeted secondary structure as WT Der p 2, except for mutant K96A which resulted in a misfolded protein (Fig. 1b). Interestingly, mutations to several neighbouring residues (Y90A, W92A, N93A, I97A, K100A and E102A) did not show structural changes compared to wild type Der p 2 (Supplementary Figure 1). We nevertheless retained mutant K96A in the panel as a means of studying the effects of protein unfolding on IgE binding.

### IgE-reaction profiles of dust mite allergic patients

**Figure 2:**
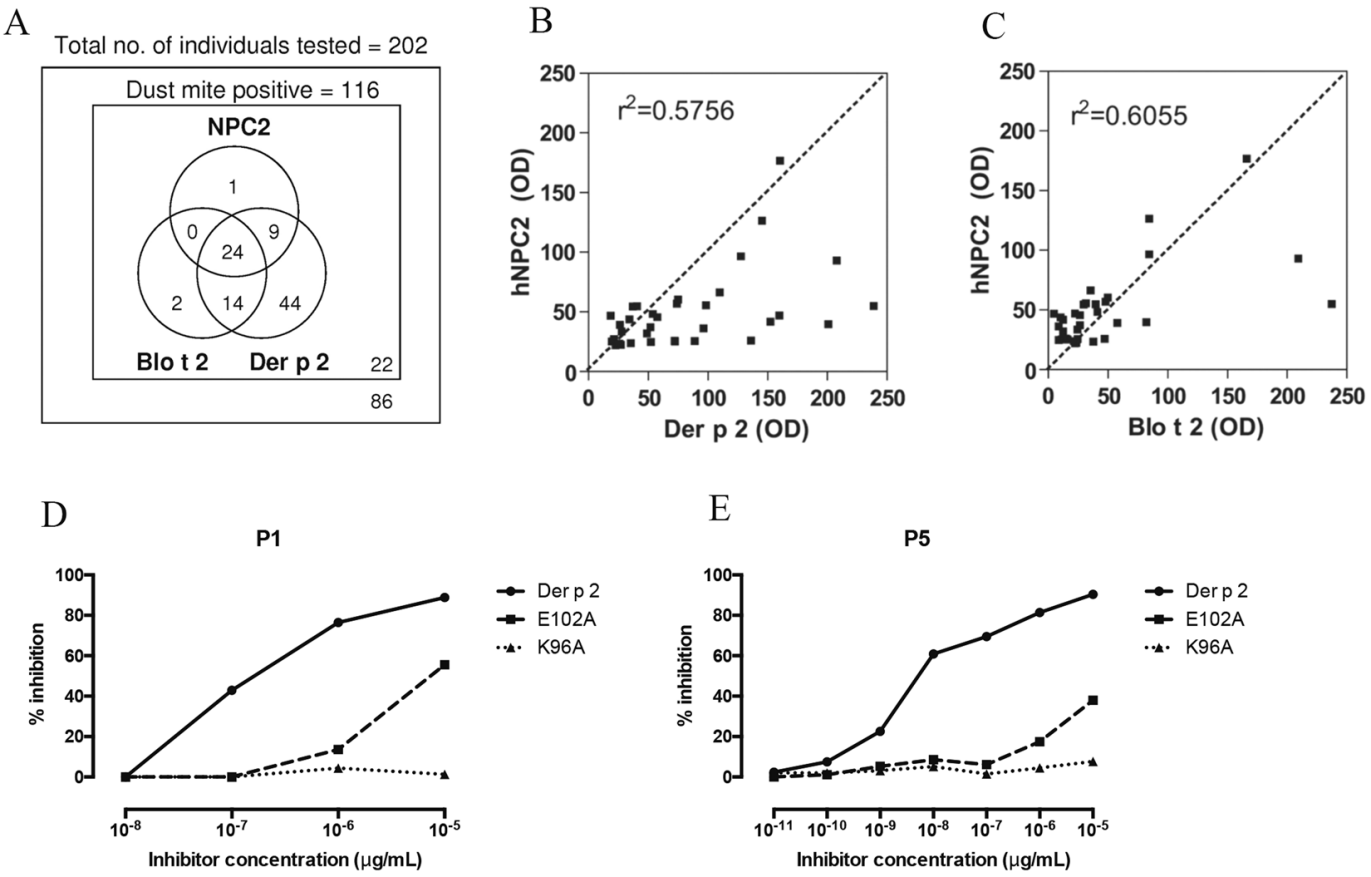
(**a**) Venn diagram (not to scale) of number of allergic individuals with IgE reactivity to Der p 2, NPC2 and Blo t 2 among individuals with allergic symptoms. IgE reactivity was measured using an immuno dot blot assay. Biplots of IgE reaction between (**b**) Der p 2 and hNPC2 or (**c**) Blo t 2 and hNPC2, among house dust mite allergic individuals was assayed using immuno dot blots and measured as optical density (OD). The coefficients of correlation are presented on each graph (p < 0.01). (**d**,**e**) Inhibition of IgE antibody binding from two representative allergic individuals between WT Der p 2 and its mutants are shown. Data presented are mean and standard deviations of two experiments.

The IgE binding of the different mutants generated were tested by dot-blots using serum from allergic individuals. Of the 202 allergic sera samples, 116 (57%) demonstrated IgE binding to crude dust mite extracts of *Dermatophagoides pteronyssinus* and *Blomia tropicalis*, the two predominant dust mite species in Singapore^16^. Among the dust mite allergic individuals, 78% had Der p 2-specific IgE (Fig. 2a). We observed that 34 individuals had IgE binding to hNPC2 of which 33 had IgE binding to another structurally similar allergen (Der p 2 alone, or both Der p 2 and Blo t 2). However, only low to moderate concordance between the individuals’ specific IgE responses to Der p 2 and hNPC2, and between Der p 2 and Blo t 2 were observed (Fig. 2b,c). We next tested the IgE binding of the 21-single site-directed alanine mutants of Der p 2 that we generated using serum from dust-mite allergic individuals. In this large population screen involving 116 dust-mite positive individuals, we observed that five mutants, N10A, E25A, K77A, E102A and K96A showed a consistent reduction in IgE binding across all sensitization groups (Table 1), and therefore constitute the major conformational IgE epitopes of Der p 2 in our study population.

### Specific IgE binding to site directed mutants of Der p 2 in five allergic individuals

Sera from five allergic patients, from whom sera was abundant and fresh blood samples were available for cellular studies were then tested the IgE binding to WT Der p 2, WT Blo t 2, hNPC2 and 21 Der p 2 mutants. All individuals showed almost negligible levels of IgE reactivity to hNPC2 (Fig. 1a). Each individual displayed a unique IgE binding profile to the different Der p 2 mutants tested. Mutants K96A and E102A showed consistent reduction in IgE binding in all patients (Table 2). The misfolded Der p 2 (K96A) showed the lowest amount of IgE binding in all individuals, averaging 89% reduction compared to WT Der p 2 (Fig. 1a). In addition, an additional fifty-nine Der p 2-sensitized patients were assayed for IgE binding to the wild type Der p 2, human NPC2, wild type Blo t 2, and Der p 2 alanine mutants, E25A, K96A and E102A using immuno-dot blots (Supplementary Table 1). The data was presented as percentage of IgE reactivity, with the IgE reactivity of Der p 2 normalized to 100%. Data from this larger population screen was concordant to the IgE reactivity data measured using ELISA (Fig. 1a), where mutant K96A of Der p 2 showed the lowest IgE binding capacity among Der p 2 sensitized individuals. Mutant E102A also showed reduced IgE binding compared to Der p 2, although to a lesser extent. Differences between IgE binding to wild type Der p 2 and mutant K96A or mutant E102A were statistically significant (p < 0.0001).

**Table 2:** Site directed mutants with < 75% IgE binding compared to WT Der p 2 in five selected dust-mite allergic individuals.

Patient	Mutants with <75% IgE binding compared to WT Der p 2
P1	**K96A, E102A**
P2	**K96A, E102A**
P3	L61A, K77A, **K96A, E102A**, N114A
P4	E25A, K66A, H74A, K77A, **K96A**, K100A, **E102A**, N114A, R128A
P5	**K96A, E102A**

Mutants in bold showed reduced IgE binding in all allergic individuals tested.

An inhibition ELISA was next used to investigate the lack of serum IgE reactivity to mutants E102A and misfolded Der p 2. IC_50_ of mutant E102A was at least 4-fold higher, while for K96A it was 16-fold higher compared to WT Der p 2 in all allergic individuals tested (Table 3, Fig. 2d,e).

**Table 3:** Inhibition of IgE binding to immobilized WT Der p 2, by addition of mutant K96A or E102A.

Patient	IC_50_ Der p 2 (ng/mL)	IC_50_ K96A (ng/mL)	IC_50_ E102A (ng/mL)	IC_50_ K96A/WT Der p 2	IC_50_ E102A/WT Der p 2
P1	1.70E-04	1.10E-01	7.00E-03	664.88	41.5
P2	3.20E-04	NA	1.50E-03	NA	4.59
P3	7.90E-03	NA	4.60E-01	NA	57.93
P4	1.10E-03	1.90E-02	1.10E-02	16.43	9.49
P5	4.50E-06	1.10E-02	2.20E-01	23949.96	48145.67

IC_50_ refers to the concentration of allergen that inhibits 50% of the IgE from binding to immobilized WT Der p 2. The fold difference between the concentrations of mutant and wild type protein to cause 50% inhibition is shown.

### Mouse IgG antibodies raised against mutants E102A and K96A are able to block allergic individuals’ IgE binding to WT Der p 2

**Figure 3:**
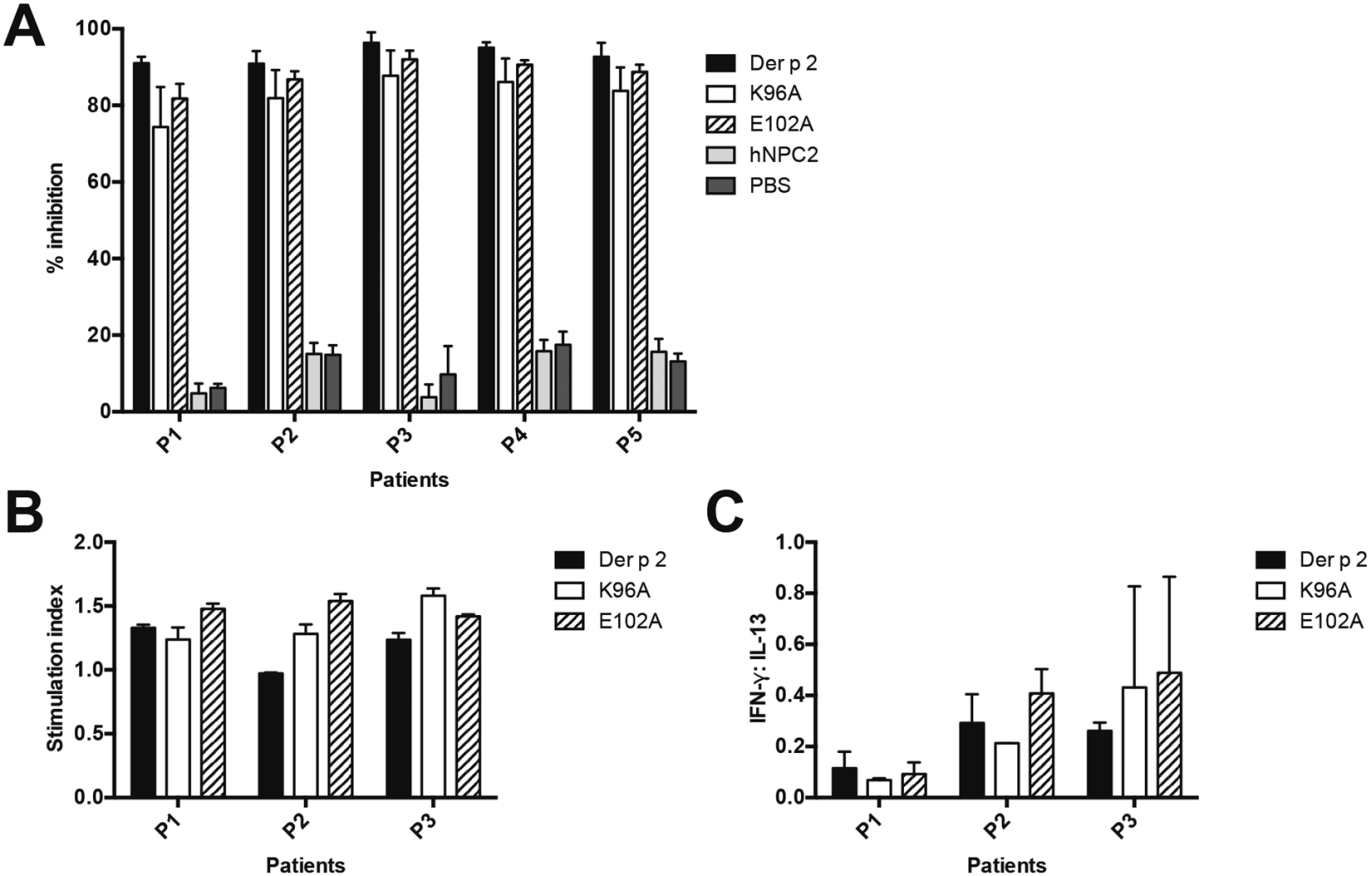
(**a**) Percentage of inhibition of human serum IgE binding to WT Der p 2 after pre-incubation with immunized mouse serum. Each group of mice was immunized with either wild type Der p 2 (WT), mutant K96A, mutant E102A, hNPC2 or PBS. All experiments were performed in duplicates, and standard deviations of experiments are shown. (**b**) Stimulation index of individual patient PBMCs stimulated with WT Der p 2, mutant K96A or E102A. PBMCs from Der p 2 allergic individuals were stimulated with the relevant proteins and PBMC proliferation was measured at day 6 using the BrdU kit. Averages and standard deviation of duplicate experiments are shown. (**c**) Cytokine secretion profiles of the same patients tested in (**b**) are shown. Cytokines IFN-γ and IL-13 were used as markers of Th1 and Th2 subtypes respectively. The results are presented as average and standard deviations of two independent experiments.

One of the first indications of successful immunotherapy in humans is the increase in antigen specific IgG levels, which competes and blocks IgE-binding to the allergen^17^. To investigate the effectiveness of mutants E102A and K96A as potential hypoallergens, specific IgG antibodies were raised in mice, by immunization with WT or mutants of Der p 2, and inhibition of human IgE binding to WT Der p 2 was assessed using ELISA (Fig. 3a). Mouse IgG antibodies against WT Der p 2 showed almost complete inhibition of human IgE binding to Der p 2 at > 90% inhibition. Similar inhibitory effect was also observed when IgG antibodies against mutants E102A and K96A were used to inhibit IgE binding to Der p 2, suggesting that both mutants were capable of generating blocking antibodies. IgG antibodies from mice immunized against hNPC2 and PBS could not inhibit the binding of allergic individuals’ IgE to Der p 2 (Fig. 3a).

### Der p 2 mutants lacking IgE reactivity retained their T-cell epitopes

Next, we evaluated the ability of mutants E102A and K96A to stimulate the proliferation of peripheral blood mononuclear cells (PBMCs) from dust-mite allergic individuals. Both E102A and K96A were able to stimulate PBMC proliferation comparable to WT Der p 2 in three allergic individuals tested, indicating the Der p 2 mutants retained functional T-cell epitopes (Fig. 3b). To determine the subtype of T-cells that proliferated, we measured the concentrations of IFN-γ (Th1 cytokine) and IL-13 (Th2 cytokine) released into the cell culture supernatant (Fig. 3c). Additional T-cell proliferation studies were performed on patients showing similar results, with mean stimulation index ranging from 1.5–2.1 and the mean IFN-gamma:IL13 ratio ranging from 0.2–0.8. No significant differences were observed between the wild type Der p 2 allergens and the comparative mutants. As the Th1:Th2 cytokine ratio when stimulated with WT Der p 2 or its mutants were not statistically significant, a distinction of a dominant T-cell subtype could not be determined.

## Discussion

Der p 2 is regarded as an important allergen worldwide, and it is therefore of clinical importance to understand its epitopes, and to evaluate the potential hypoallergen vaccine candidates of this protein. Conventional therapy for allergies such as the use of anti-cytokines, anti-IgE or corticosteroids are only able to alleviate the symptoms experienced, but do not confer long term protection^17^. Immunotherapy represents a form of curative treatment for allergies as it causes immune deviation or tolerance in a patient’s immune mechanism^8^. Although natural allergens were initially used for immunotherapy, they could potentially cause anaphylactic shock in some patients due to the presence of their IgE-binding epitopes. T-cells have been shown to play an important role in the tolerance mechanism for immunotherapy^18^. Therefore, to provide safer therapeutic reagents, immunotherapy molecules which lack the IgE epitopes (hypoallergens) but retained T-cell proliferation are generated^19^.

Single alanine mutants of surface exposed amino acids of Der p 2 were generated to evaluate their contribution to the IgE reactivity of Der p 2. The IgE epitopes of Der p 2 were mapped in 116 dust mite-sensitized individuals from Singapore. Five mutants, N10A, E25A, K77A, K96A and E102A showed a consistent reduction in IgE binding across all sensitization groups in our study, and therefore constitute major IgE epitopes of Der p 2 in our study population. Of these, two mutants (K96A and E102A) have been identified earlier as IgE epitopes of Der p 2 using a different approach^12^. Our study showed that alanine mutation of K96 resulted in a drastic change in the secondary structure of the protein, from a β-sheeted structure to a misfolded structure, based on the circular dichroism (CD) spectra data, indicating that it is a crucial residue in the refolding of Der p 2. No significant changes in secondary structures were observed when residues adjacent to K96 were mutated (Supplementary Figure 1). In a study involving a cherry allergen, Pru av 1, mutation of a single amino acid residue also resulted in a misfolded protein structure that showed nearly complete loss of IgE binding^20^. However, not all amino acid residues seem to be critical for protein refolding, as seen from our data on mutants such as N10A, E25A, K77A and E102A, which retained their secondary protein structure comparable to the wild type protein. In these cases, the reduction in IgE binding could be a result of the local changes in surface accessibility or charge.

Two mutants, K96A and E102A to further characterize their hypoallergen properties. Both mutants showed reduced IgE binding in all patients when compared to WT Der p 2. Both mutants were also able to inhibit serum IgE of allergic individuals from binding to WT Der p 2 allergen in inhibition ELISA assays, albeit to different degrees. The IC_50_ ratio of mutant:native was higher when K96A was used as the inhibitor compared to E102A, suggesting that some IgE binding epitopes were still present on mutant E102A. In contrast, the majority of the IgE epitopes of mutant K96A may have been destroyed due to the protein misfolding.

The production of allergen-specific IgG antibodies has been reported to be among the first signs of successful immunotherapy^21,22^. There have been several proposed mechanisms by which allergen specific IgG play a role in immunotherapy. First, allergen specific IgG ‘blocks’ IgE from binding to the allergen by direct competition, resulting in the lack of IgE cross-linking, reducing the release of mediators from mast cells and basophils^23,24^. Alternatively, the IgG generated may interfere with the IgE-allergen complex which binds to the low affinity IgE receptor on antigen presenting cells, CD23, which facilitates the allergen presentation to T-cells^25,26^. Due to the non-reversible nature of immunoglobulin gene recombination, cells which already produce IgE antibodies will remain doing so, even after immunotherapy. Hence, no change in the amounts of IgE would be observed when pre- and post-treatment levels are compared^27^. The increase in IgG production causes an increase in the IgG:IgE ratio, which then results in the immunological changes post-immunotherapy. In pollen allergy, immunotherapy shows a seasonal rise in IgG, particularly IgG_4_^28^ while gradually attenuating the seasonal rise in IgE^21^.

In this study, sera from mice immunized with E102A or K96A were able inhibit human IgE binding to WT Der p 2 at similar intensity, which was only slightly lower than homologous inhibition using WT Der p 2. Since the mutants lack the IgE binding epitopes, this inhibition could be due to steric hindrance, and not direct competition in IgE binding. The production of blocking IgG antibodies have also been observed for other hypoallergen vaccine candidates from house dust mites^29,30^ and birch pollen allergen, Bet v 1^31^.

Although much emphasis has been placed on the production or expression levels of cytokines, which are indicative of the T-helper subtype following allergen stimulation, results from different studies have been conflicting. In this study, we observed that although mutants E102A and K96A was able to stimulate PBMC proliferation, a clear distinction in T-helper subtype could not be made. Similarly, Klimek *et al*.^32^ reported that birch pollen allergoid used for immunotherapy failed to change cytokine expression for IL-4, IL-5, IL-10 or IFN-γ in cell cultures but found expected increases in cytokines in nasal secretions on the immunotherapy patients. In a study of grass pollen immunotherapy, seasonal increase in the ratio of IFN-γ to IL-5 was found in the nasal mucosa, but not in the allergen-stimulated T-cell cultures^33^. Nevertheless, other studies have shown enhanced IFN-γ and reduced IL-4 production after allergen stimulation^34,35^. It appears that the switch from Th2 to Th1 cytokines is more prominent in later stages of immunotherapy, and less pronounced in a T-cell culture, as the cells are in contact with the allergen for a shorter time period.

Of the two hypoallergen candidates evaluated, the misfolded K96A showed almost negligible serum IgE binding but was able to induce blocking IgG antibody production that inhibited the binding of allergic-patient serum IgE to WT Der p 2. In addition, the T-cell epitopes of K96A remained intact as it was capable of stimulating PBMC proliferation. These characteristics suggests that mutant K96A is promising hypoallergenic vaccine candidate and should be further tested in pre-clinical trials as specific immunotherapy against house dust mite allergy.

## Methods

### Subcloning and site directed mutagenesis

DNA encoding for Der p 2 were amplified from cDNA of *Dermatophagoides pteronyssinus* using primers containing *BamH* I and *Eco R* I restriction sites. The DNA insert was ligated into a modified pET-32a plasmid (Novagen) and transformed into DH5-α competent cells. Colonies were screened by PCR, and the insert sequence was verified by DNA sequencing (Big Dye v3.1; Applied Biosystems). Mutant constructs were generated using the Quikchange® kit (Statagene) with primers containing mismatches coding for alanine. Correct substitutions were verified by DNA sequence analysis. Mutated DNA insert was sub-cloned in the same manner as WT Der p 2.

### Expression and purification of wild type and mutant Der p 2

Plasmid containing DNA insert of WT or mutant Der p 2 was transformed into *E*. *coli* (BL21, DE3) for protein expression. Cultures were induced overnight with 1.0 mM IPTG at 20 °C. The protein was expressed as a His-tagged soluble protein and purified using a Ni-NTA resin (Novagen) under denaturing conditions. Recombinant proteins were refolded by rapid dilution into 50 mM sodium acetate, pH 4.6 at 4 °C and concentrated using Amicon® Stir Cell (YM3, Millipore).

### Circular dichroism (CD) spectropolarimetry

CD spectra was acquired using a J-180 Spectropolarimeter (Jasco) using a 1 mm path length quartz cuvette. The spectra were recorded at the resolution of 0.1 nm and averaged for 10 scans (50 nm/min) from 190 to 260 nm.

### Ethics approval for serum samples and mice immunizations

Consecutive serum samples from patients from Singapore with clinical symptoms of allergies were used. Written informed consent were obtained from all participants to obtain blood samples. The human and animal studies were reviewed and approved by the Institutional Review Board of the National University Hospital, the Animal Research Ethics Committee of the National University of Singapore and the Hospital Ethics Committee of the KK Women’s and Children’s Hospital and Singapore General Hospital. All experiments were performed in accordance with relevant guidelines and regulations of the institutions and committees indicated above.

### Immuno dot-blot

One microgram of crude allergen extract or recombinant proteins were dotted on nitrocellulose membranes (BIO-RAD Laboratories, USA), air dried and blocked with PBS-0.1% Tween-20. Membranes were incubated with serum from allergic individuals overnight at 4 °C, followed by alkaline phosphatase conjugated anti-human IgE (Sigma Aldrich, USA) for 2 hrs. Membranes were developed with NBT/BCIP (nitroblue tetrazolium/5-bromo-4-chloro-3-indolyl-phosphate) (Promega). Spot intensities were measured using an image analysis software (Microimage v.3.01, Germany). Spot intensities (range, 0–255) were normalized by subtracting the background. Intensities greater than 2 SDs above the mean negative sera responses were considered positive. For comparison, a standard curve using the human serum IgE standard (75/502), purchased from the National Institute for Biological Standards and Control (NIBSC), United Kingdom, was used.

### Specific IgE binding ELISA

WT or mutant Der p 2 were coated overnight at 4 °C onto microtiter plates (Nunc) at 250ng of protein per well. After blocking, wells were incubated with patients’ sera diluted 1:10 or 1:5, and incubated at room temperature (RT) for 2.5 hrs. After washing, plates were incubated with biotin conjugated anti-human IgE monoclonal antibody (BD-Pharmingen, USA), followed by avidin conjugated HRP (BD-Pharmingen, USA) for 30 min. Plates were developed by adding TMB (3,3′,5,5′-Tetramethylbenzidine) substrate (Sigma Aldrich, USA). The colour reaction was stopped after 30 mins using 1 M HCl. Absorbance was measured at 450 nm using a microplate reader.

### Inhibition ELISA

Inhibition ELISAs was performed according to the ELISA protocol described, except that the sera used were pre-absorbed with 1 × 10^−4^ to 1 × 10^−11^ μg/mL of recombinant allergen. The degree of cross-reactivity was calculated by the percentage of inhibition based on the following formula: [(I_u_ − I_i_)/(I_u_ − B)] × 100%, where I_u_ represents the reaction in the absence of inhibitor protein, I_i_ the reaction at a particular inhibitor concentration, and B represents background intensity when the allergen was incubated with blocking solution instead of sera.

### Peripheral blood mononuclear cells (PBMC) proliferation and cytokine expression

Fresh blood was collected from three allergic individuals and PBMCs were isolated using the Ficoll-Hypaque gradient centrifugation. PBMCs were cultured in RPMI with 10% FBS, 50 units/mL penicillin, 50 μg/mL streptomycin and 2mM L-glutamine in 96-wells plate at 10^5^ cells per well. WT or mutant Der p 2 were added to the cell suspension (final concentration of 10 μg/mL), and incubated for 6 days at 37 °C under 5% CO_2_. Cell proliferation was measured using the Bromodeoxyuridine colorimetric method using the manufacturer’s protocol (Roche Applied Sciences). Proliferation is reported as stimulation index, calculated by the formula S/C (S, absorbance of stimulated cells; C, absorbance of non-stimulated cells). To measure cytokines, supernatants of the cell culture on day 6 were harvested, and cytokine concentrations were determined using human Th1/Th2 Bioplex cytokine assay (Biorad) according to the manufacturer’s protocol.

### Mouse Immunization

Six-week old female Balb/c mice (n = 4) were injected intra-peritoneally with 10 μg of purified WT or mutant Der p 2, or hNPC2 with 1.25 mg/mL aluminium hydroxide gel (Alum, Sigma Aldrich, USA) every 14 days. PBS buffer containing Alum was similarly injected into another group of 2 mice as controls. Pre- and post-immune blood samples after the 6th injection was drawn by orbital bleeding and stored at −20 °C until use.

### Inhibition of human IgE binding to Der p 2 by specific mouse IgG antibodies

WT Der p 2 was coated overnight at 4 °C onto Maxisorp ELISA plate (Nunc) at 250ng of protein per well. After a washing and blocking step, wells were incubated with 1:10 diluted mouse sera for 2 hrs. Next, wells were incubated with diluted patients’ sera for another 2 hrs. The remaining steps were similar to that described in the IgE binding ELISA. Percentage inhibition of human IgE binding to Der p 2 was calculated using the following formula: % inhibition of IgE binding = 100 − (A − B) × 100%. A and B represent the absorbance values after pre-incubation with serum from immunized mouse and control mouse respectively.

### Statistical analysis

Statistical analysis was carried out using GraphPad Prism. Differences in IgE binding between wild type Der p 2 and mutant allergens of individual patients was calculated using two-way ANOVA with Dunnette’s multiple comparison test at a significance of p < 0.0001. Comparisons between allergen levels in the dust samples were performed using paired *t*-test.

## Electronic supplementary material

Supplementary Information
