# Supplementary material for: Conformational IgE Epitope Mapping of Der p 2 and the Evaluations of Two Candidate Hypoallergens for Immunotherapy
Source: Sci Rep. 2018 Feb 21;8:3391. doi: 10.1038/s41598-018-21792-1 (PMC5821840; doi:10.1038/s41598-018-21792-1)
Supplement: Supplementary file 1 — Supplementary Information [file 41598_2018_21792_MOESM1_ESM.pdf]

Conformational IgE Epitope Mapping of Der p 2 and the Evaluations of Two Candidate Hypoallergens for Immunotherapy

Short title: Der p 2 IgE epitope mapping and studying two hypoallergens

Kavita Reginald[1, 2] and Chew Fook Tim[3]*

[1]Research Centre for Biomedical Sciences, Sunway University, Bandar Sunway 47500, Selangor, Malaysia

[2]Department of Biological Sciences, Sunway University, Bandar Sunway 47500, Selangor, Malaysia

[3]Allergy and Molecular Immunology Laboratory, Department of Biological Science, National University of Singapore, 117543 Singapore

Corresponding author:

Dr. Chew Fook Tim, Allergy and Molecular Immunology Laboratory, Lee Hiok Kwee Functional Genomics Laboratories, Department of Biological Sciences, 14 Science Drive 4, National University of Singapore, 117543 Singapore, Tel. +65-65161685; Fax +65-68722013; E-mail: dbscft@nus.edu.sg

Supplementary Table 1. IgE reactions of fifty-nine Der p 2-sensitized individuals to selected allergens assayed using immune-dot blots. IgE reactions have been normalised to individual patient's Der p 2 reactivity (set at 100%). Differences between IgE binding to wild type Der p 2 and mutant K96A or mutant E102A was statistically significant (one-way ANOVA, $p<0.0001$).

	Der p 2		NPC2		Blo t 2		E25A		K96A		E102A	
	Average	Std. Dev.	Average	Std. Dev.	Average	Std. Dev.	Average	Std. Dev.	Average	Std. Dev.	Average	Std. Dev.
P0001	100.00	0.00	2.49	0.17	0.37	0.88	35.66	4.45	0.00	5.58	36.92	1.62
P0002	100.00	0.00	44.11	0.22	30.90	2.23	25.52	0.54	0.00	2.00	86.29	4.15
P0003	100.00	0.00	20.48	6.39	36.97	2.03	70.86	8.59	2.24	2.82	75.26	6.04
P0004	100.00	0.00	22.68	0.58	0.00	0.20	59.38	3.02	0.00	1.97	41.25	7.94
P0005	100.00	0.00	21.35	1.01	0.00	1.09	45.83	3.17	0.00	4.32	36.37	8.98
P0006	100.00	0.00	23.90	0.79	0.00	0.58	33.69	1.48	0.00	1.95	63.99	7.35
P0007	100.00	0.00	75.04	1.65	22.60	0.49	85.39	0.81	0.00	4.14	66.43	0.01
P0008	100.00	0.00	72.33	0.22	11.94	1.07	30.66	1.44	0.00	3.47	28.31	3.91
P0009	100.00	0.00	68.06	1.18	40.73	0.45	35.99	6.95	4.99	0.15	49.63	3.19
P0010	100.00	0.00	47.85	1.09	0.00	0.72	77.80	2.84	11.80	0.49	12.47	0.73
P0011	100.00	0.00	45.90	0.66	0.00	1.59	57.05	0.78	7.50	0.61	0.00	3.43
P0012	100.00	0.00	40.61	1.71	0.00	0.24	19.86	0.39	0.00	3.67	74.18	6.06
P0013	100.00	0.00	38.18	2.45	0.00	5.53	-0.45	1.70	0.00	9.52	53.02	1.00
P0014	100.00	0.00	65.81	1.56	53.44	0.65	62.65	3.59	3.23	0.26	84.03	4.78
P0015	100.00	0.00	8.09	0.94	0.00	1.78	73.24	0.72	0.00	3.93	69.29	15.18
P0016	100.00	0.00	65.28	5.12	0.00	3.39	0.00	1.01	0.00	1.38	55.03	2.70
P0017	100.00	0.00	25.22	0.52	27.15	3.59	35.93	1.87	9.07	3.42	44.07	5.44
P0018	100.00	0.00	46.35	1.84	15.75	1.73	15.66	1.64	0.00	1.71	76.64	4.77
P0019	100.00	0.00	47.94	5.13	11.37	1.06	13.72	8.76	1.60	0.20	61.06	2.19
P0020	100.00	0.00	17.31	2.34	46.57	2.47	68.62	2.24	5.34	4.27	64.80	4.62
P0021	100.00	0.00	62.67	1.04	32.55	0.95	14.45	0.79	18.82	5.21	37.26	7.90
P0022	100.00	0.00	32.07	4.57	11.76	7.40	19.91	2.92	0.00	0.62	31.89	9.89
P0023	100.00	0.00	16.31	1.15	23.99	3.33	3.32	1.17	0.00	0.04	47.63	1.17

P0024	100.00	0.00	2.47	1.86	46.05	3.10	56.42	0.92	1.63	2.65	34.03	0.36
P0025	100.00	0.00	10.69	14.11	22.01	1.25	74.59	0.12	14.38	1.16	78.54	4.57
P0026	100.00	0.00	20.26	2.73	23.60	2.37	44.37	4.00	0.00	5.05	41.55	0.66
P0027	100.00	0.00	8.34	14.29	25.11	8.43	0.00	19.51	9.41	0.36	85.20	54.78
P0028	100.00	0.00	0.00	1.84	0.00	4.16	53.35	0.24	0.00	1.45	7.82	6.78
P0029	100.00	0.00	43.01	1.33	100.00	1.47	60.62	0.51	2.44	1.31	0.00	4.33
P0030	100.00	0.00	16.14	4.75	74.77	4.85	57.79	0.92	8.90	0.08	79.53	7.33
P0031	100.00	0.00	36.75	1.60	22.93	1.58	55.90	2.45	10.59	1.51	76.26	6.59
P0032	100.00	0.00	18.12	1.92	2.51	1.29	54.90	6.10	0.00	1.11	70.04	6.21
P0033	100.00	0.00	7.67	1.02	28.74	2.82	81.51	1.15	10.81	4.37	42.59	0.76
P0034	100.00	0.00	49.20	2.72	0.00	0.26	0.00	4.50	9.89	5.18	88.06	6.25
P0035	100.00	0.00	71.04	3.21	52.50	5.78	44.96	5.58	29.69	0.52	20.20	4.07
P0036	100.00	0.00	0.71	0.66	22.15	1.21	8.30	0.42	8.26	0.28	39.06	4.73
P0037	100.00	0.00	38.63	1.55	33.75	4.62	44.39	0.42	10.80	0.55	0.00	0.08
P0038	100.00	0.00	19.85	2.77	20.34	2.60	53.27	2.21	1.83	4.88	2.00	1.07
P0039	100.00	0.00	0.00	4.51	8.60	2.86	99.34	0.22	27.03	2.60	0.00	0.71
P0040	100.00	0.00	21.42	6.47	100.00	0.81	100.00	1.33	0.00	1.09	46.62	0.38
P0041	100.00	0.00	18.89	3.99	11.28	1.07	74.15	15.52	9.18	2.57	27.91	0.40
P0042	100.00	0.00	7.91	1.18	62.94	3.40	3.21	4.97	0.00	0.42	0.00	2.67
P0043	100.00	0.00	23.02	2.78	67.20	5.27	52.48	4.18	17.29	0.09	32.86	18.59
P0044	100.00	0.00	0.00	4.92	17.50	5.77	19.46	3.34	0.00	0.45	60.91	17.09
P0045	100.00	0.00	27.76	2.05	87.68	7.42	67.07	11.94	0.00	0.35	25.28	6.14
P0046	100.00	0.00	0.00	13.60	32.00	5.97	67.59	4.04	10.02	6.04	22.19	5.34
P0047	100.00	0.00	8.57	9.19	44.70	0.24	87.43	6.39	5.97	3.42	43.24	2.11
P0048	100.00	0.00	19.81	0.78	4.55	1.36	55.47	4.70	10.01	0.37	87.07	0.41
P0049	100.00	0.00	0.00	0.95	0.00	2.80	97.86	5.38	0.00	2.26	85.80	5.85
P0050	100.00	0.00	77.57	3.30	55.03	2.91	57.82	7.40	2.66	0.96	0.00	1.44
P0051	100.00	0.00	35.69	4.11	65.93	4.84	77.50	8.16	8.27	3.04	57.74	6.83
P0052	100.00	0.00	27.48	0.29	11.04	0.24	63.80	0.12	0.00	1.02	23.56	8.54
P0053	100.00	0.00	22.70	0.79	8.03	0.57	66.23	0.95	0.00	1.07	27.97	8.85
P0054	100.00	0.00	29.75	2.25	19.28	3.66	100.00	1.31	19.92	0.61	62.59	1.66
P0055	100.00	0.00	33.91	1.89	16.17	2.28	98.40	14.43	9.36	1.07	77.91	3.49

P0056	100.00	0.00	0.00	4.54	0.00	6.03	3.22	3.35	0.00	1.65	0.00	1.95
P0057	100.00	0.00	11.68	0.23	3.37	4.32	58.22	3.95	6.99	0.35	35.48	1.20
P0058	100.00	0.00	27.60	0.92	9.12	0.12	59.52	8.71	17.35	2.13	84.67	9.65
P0059	100.00	0.00	29.56	4.80	14.84	3.53	93.91	10.71	20.97	1.63	64.06	17.04

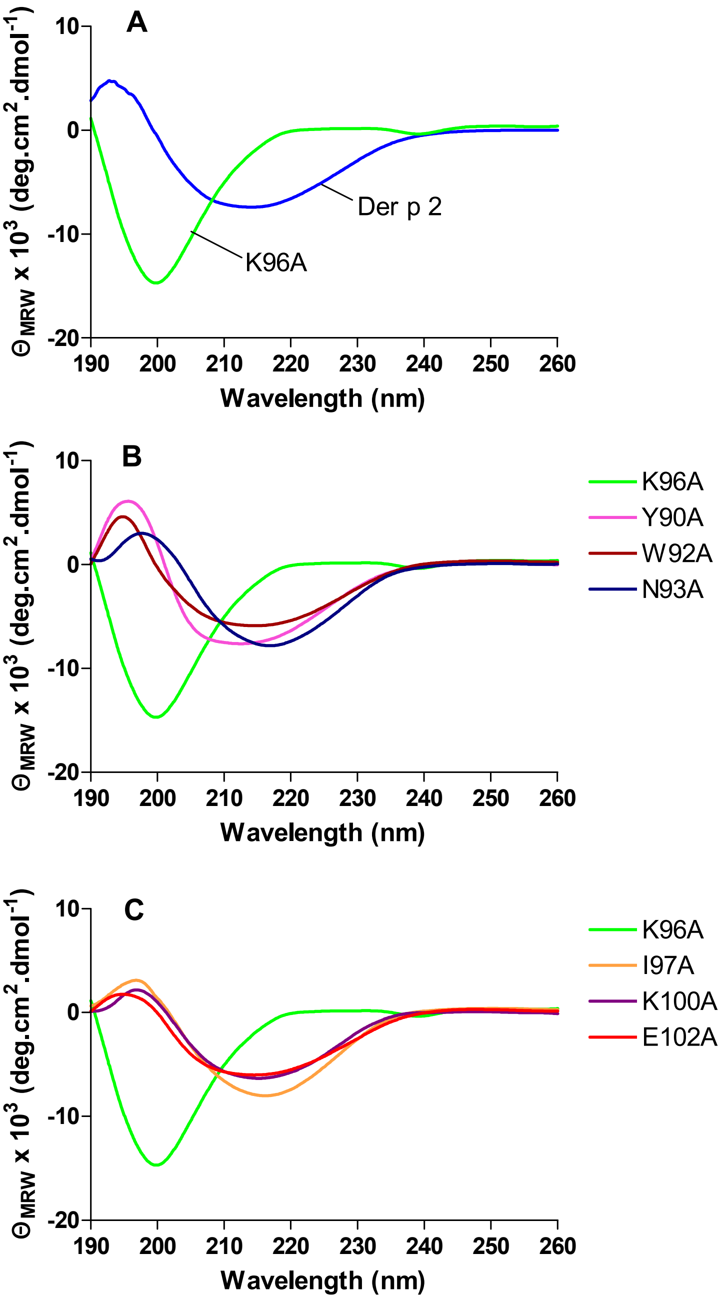

Supplementary Figure 1. Circular dichroism (CD) spectra of WT Der p 2 and alanine mutants of Der p 2. (A) Far-UV CD spectra of WT Der p 2 (blue line) and K96A mutant (green line) were recorded at room temperature, and averages of 10 scans are presented. WT Der p 2 shows a typical spectrum of a β-sheeted protein. (B-C) CD spectra of mutants Y90A, W92A, N93A, I97A, K100A and E102A were compared to the spectra of unfolded Der p 2.
